# JAK inhibitors for the treatment of rheumatoid nodules

**DOI:** 10.1016/j.jdcr.2025.02.036

**Published:** 2025-03-14

**Authors:** Brianna Bulbin, Neil Kramer, Elliot David Rosenstein, Rachel Kushner Rosenstein

**Affiliations:** a Hackensack Meridian School of Medicine, Hackensack University Medical Center, Nutley, New Jersey; b Division of Rheumatology, Department of Internal Medicine, Institute for Rheumatic & Autoimmune Diseases, Overlook Medical Center, Summit, New Jersey; c Division of Rheumatology, Department of Internal Medicine, Atlantic Medical Group Rheumatology, Atlantic Health System, Morristown, New Jersey; d Department of Internal Medicine and Center for Discovery and Innovation, Hackensack Meridian School of Medicine, Hackensack University Medical Center, Nutley, New Jersey

**Keywords:** cytokines, immunology, JAK inhibitors, rheumatoid arthritis, rheumatoid nodules

## Introduction

Rheumatoid arthritis (RA) is a chronic, systemic, autoimmune disease characterized by synovial inflammation which, when uncontrolled, leads to joint destruction. The most common dermatologic manifestation of RA is rheumatoid nodules, subcutaneous masses composed of palisading granulomas with a fibrinoid necrotic center, surrounded by lymphocytes and histiocytes.^1^ Palpable nodules can occur in up to 7% of RA patients at presentation and are found in up to 35% of patients during their disease course.^2^^,^^3^ Rheumatoid nodules often develop over extensor surfaces of extremities and pressure points including the fingers, hands, and elbows.^4^ They may limit joint mobility and may become inflamed or tender when traumatized which can result in ulceration and infection. Additionally, their appearance may impact patients’ quality of life. Although most often found in the skin, rheumatoid nodules can appear in viscera, most frequently the lungs.^1^ Rheumatoid nodules are notoriously refractory to traditional RA treatments and paradoxically may worsen (accelerated nodulosis) with some therapeutic agents, particularly with methotrexate, the mainstay of RA therapy.^1^^,^^5^ Although reported in RA patients in the absence of associated autoantibodies, they are most often found among patients who have elevated levels of serum rheumatoid factor and anticyclic citrullinated peptide antibodies.^1^^,^^6^

Several of the proinflammatory cytokines constitutively activated in RA can be suppressed by inhibiting the JAK/STAT pathway. There are currently 3 JAK inhibitors (JAKi), with differing JAK specificity, FDA-approved for the treatment of moderate-to-severe RA: tofacitinib (JAK1 >3), baricitinib (JAK 1 >2), and upadacitinib (selective JAK1).^7^ We describe a series of patients with RA whose refractory rheumatoid nodules showed complete or partial clearance after treatment with JAKi.

## Case series

Seven female patients with moderate-to-severe RA with refractory rheumatoid nodules were treated with JAKi: initially, 4 received tofacitinib, 2 received upadacitinib, and 1 was switched from tofacitinib to upadacitinib (Table I). Patients had been diagnosed with RA 5-41 years prior to JAKi initiation. All of these patients had active synovitis and either new or persistent rheumatoid nodules despite prior effective treatment of synovitis with conventional synthetic disease-modifying antirheumatic drugs (such as methotrexate, hydroxychloroquine, gold, leflunomide, sulfasalazine, auranofin, penicillamine) and biologic DMARDs (etanercept, adalimumab, abatacept, infliximab). None of these cases were due to accelerated rheumatoid nodulosis. Improvement in rheumatoid nodules was determined by patient report of softening of nodules and physician examination of size and number of nodules. Results were recorded and patients were followed for 1.5-9 years after initiation of the first effective JAKi.

**Table I:** Patient demographics and disease history

Age at RA diagnosis/age at initiation of JAKi (y) Sex	Antibodies	Extra-articular manifest-ations	MTX max dose (mg)	Smoking history	Rheumatoid nodule location	Prior therapies		RA duration at initiation of JAKi (y)
						csDMARDs	bDMARDs	
1. 32/66 F	RF, ACPA	none	25∗	None	olecranon digits Achilles	gold, HCQ	etanercept∗ adalimumab abatacept	34
2. 19/60 F	RF, ACPA	SS	25∗	None	olecranon digits	gold, PCM, MTX, HCQ	etanercept∗	41
3. 28/55 F	RF, ACPA	none	25∗	None	olecranon digits	gold, HCQ	etanercept∗	27
4. 56/84 F	RF	none	20∗	Former (quit >10 y)	olecranon digits	LEF∗, HCQ	infliximab abatacept	28
5. 62/72 F	RF, ACPA	SS	25∗	None	olecranon digits	SSZ, HCQ	infliximab∗	10
6. 22/50 F	RF, ACPA	none	20∗	Former (quit >10 y)	olecranon hands, digits	HCQ, LEF, AUR, PCM	etanercept adalimumab∗ infliximab	28
7. 34/39 F	RF, ACPA	SS	20∗	None	olecranon wrist (pulmonary)	MTX	etanercept abatacept∗	5

JAKi (mg)	Reason for JAKi	Extent of/time to nodule response (mo)	Other therapies	Follow-up (time since initiation of first effective JAKi)	Other treatment effects
Tofa 11	Disease activity (DA)	Resolved/5	MTX discontinued after 7 mo on Tofa	8 y	RA remission
Tofa 11† Upa 15	DA, nodulosis	Resolved/12	MTX discontinued after 2 mo on Upa	1.5 y	RA remission
Upa 15	DA, nodulosis	Resolved/12	MTX continued	3 y	RA remission
Tofa 11	DA	Softer, smaller/6	LEF stopped, MTX discontinued after 6 mo	5 y	RA remission
Tofa 11	DA	Resolved/9	MTX discontinued after 4 y	9 y	RA remission; SS improved
Tofa 11	DA	Resolved/6	MTX discontinued after 1 y on Tofa	RA recurred in 5 y, switched to Upa 15, nodules remained absent for 4 y	RA LDA
Upa 15	DA, nodulosis	Resolved/3	MTX discontinued after 6 mo on Upa	4 y	RA remission, SS improved, (pulmonary nodule resolved)

*ACPA*, Anticitrullinated protein antibody; *AUR*, auranofin; *bDMARDs*, biologic disease-modifying antirheumatic drugs; *csDMARDs*, conventional synthetic disease-modifying antirheumatic drugs; *HCQ*, hydroxychloroquine; *JAKi*, JAK inhibitor; *LDA*, low disease activity; *LEF*, leflunomide; *MTX*, methotrexate; *PCM*, penicillamine; *RF*, rheumatoid factor; *SS*, Sjögren’s syndrome; *SSZ*, sulfasalazine; *Tofa*, tofacitinib; *Upa*, upadacitinib.

∗ Indicates most recent active therapy before JAKi introduction; bDMARDs were discontinued at JAKi introduction.

† No response of nodules to Tofa after 20 months, response only after starting Upa.

**Fig 1:**
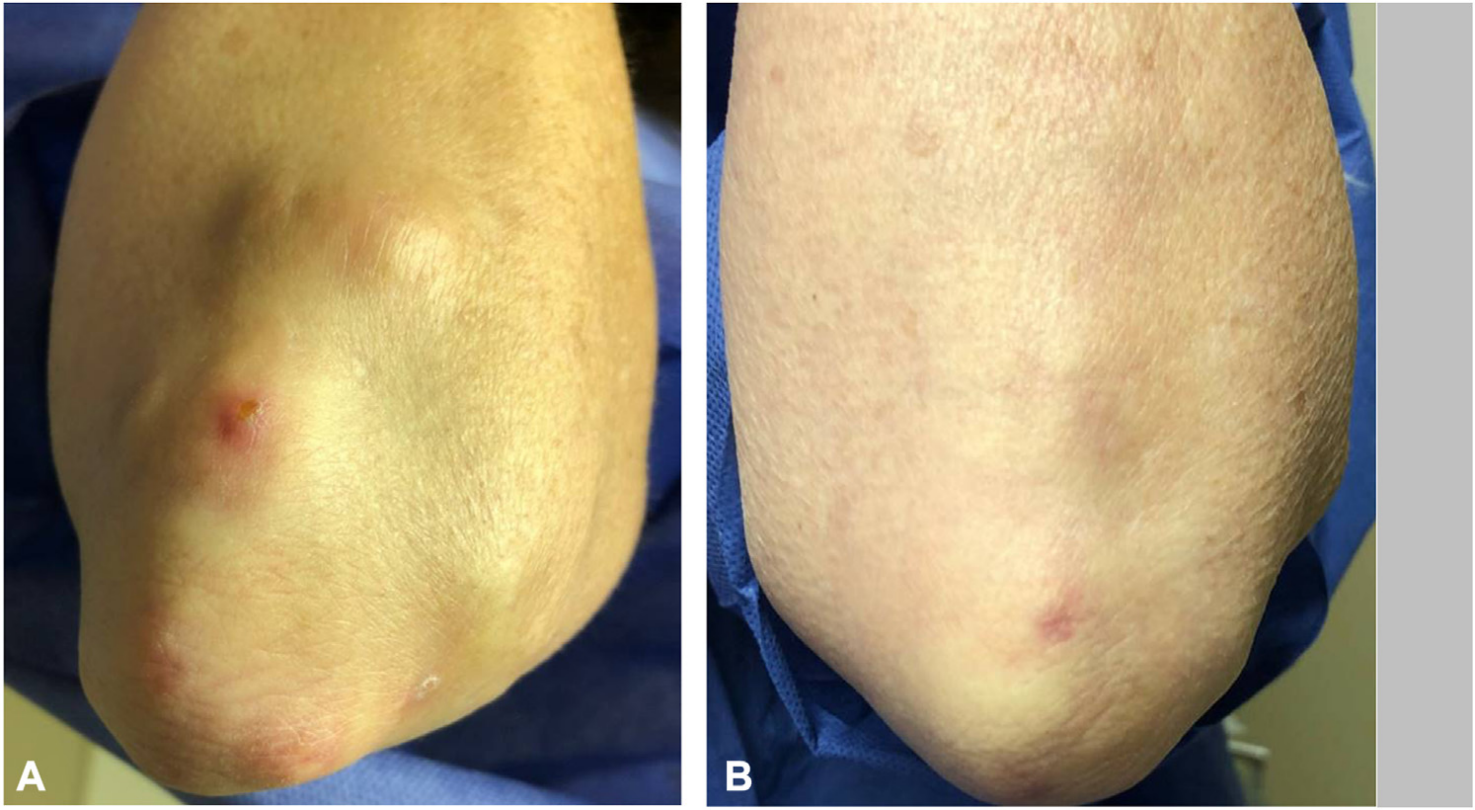
Clinical images: Patient 3. **A,** Rheumatoid nodules pre-treatment. **B,** One year after starting upadacitinib.

**Fig 2:**
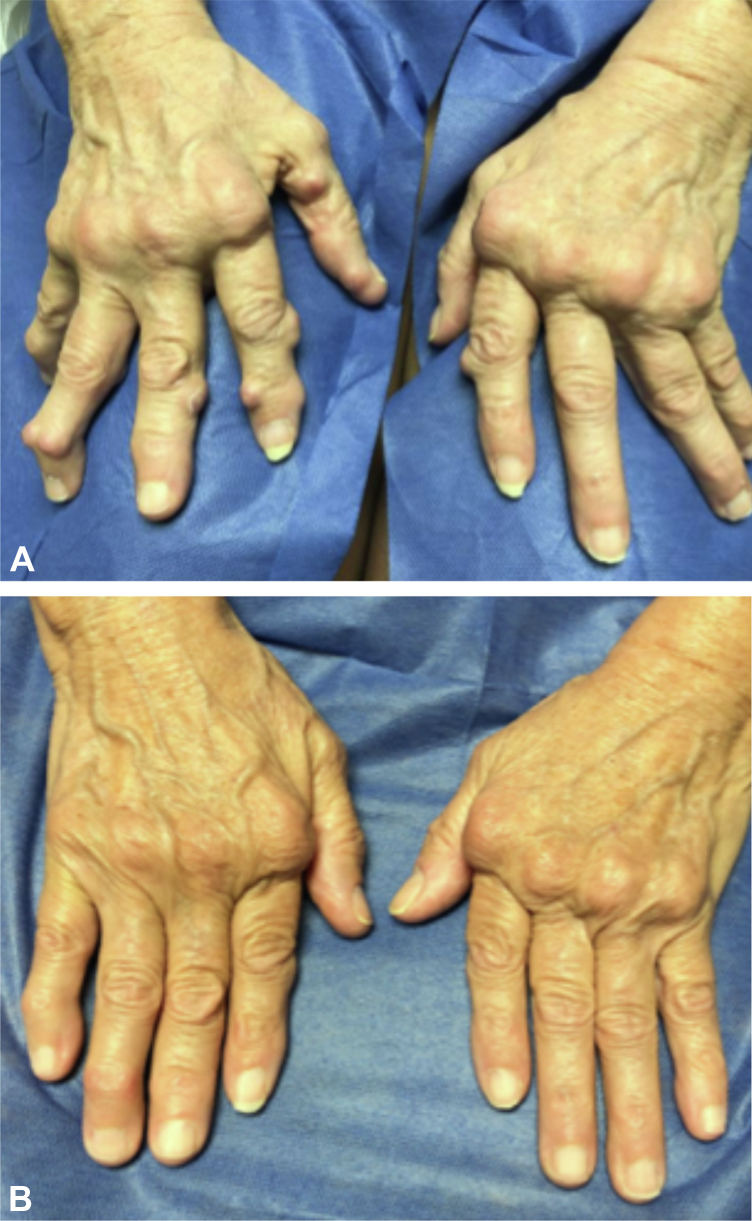
Clinical images: Patient 2. **A,** Rheumatoid nodules pretreatment. **B,** One year after starting upadacitinib.

Five patients experienced complete resolution of rheumatoid nodules (Fig 1) and one patient showed diminished size of nodules and nodule softening on her first JAKi, 3-12 months after initiating JAKi therapy. The remaining patient’s rheumatoid nodules did not improve during 20 months of tofacitinib therapy. As she continued to experience active joint involvement, she was switched to upadacitinib and noted resolution of synovitis prompting discontinuation of methotrexate after 2 months, softening of rheumatoid nodules after 4 months, and ultimately resolution of rheumatoid nodules after 12 months (Fig 2). Patients experienced RA disease remission, with 6 able to discontinue methotrexate, one of whom also discontinued leflunomide. Two patients also reported improvement in Sjogren’s syndrome and one had improvement in pulmonary nodules. Accelerated nodulosis was not seen. There were no reports of treatment-emergent infections (including herpes zoster), venous thrombotic events, cardiovascular events, or malignancies during the course of therapy.

## Discussion

Despite prior therapies for RA, all patients showed resolution or improvement in rheumatoid nodules only after treatment with JAKi, highlighting the dynamic nature of rheumatoid nodules and supporting the essential role of the JAK/STAT pathway in rheumatoid nodule pathogenesis. The patient with rheumatoid nodules for the longest duration did not experience improvement in rheumatoid nodules on tofacitinib, but noted resolution while on upadacitinib, possibly due to stronger JAK1 inhibition.

Cutaneous rheumatoid nodule response to JAKi has been previously described in an RA patient who received baricitinib.^8^ Additionally, another case described an RA patient who was seronegative for RF and ACPA who had multiple lesions including ulcerative plaques, characterized histologically as rheumatoid nodules, that responded to tofacitinib. It is however noteworthy that these lesions were atypical in appearance, size, and distribution, and the development of rheumatoid nodules in seronegative RA patients is quite unusual, suggesting the possibility that this is another necrobiotic granulomatous process.^9^ The response of cutaneous rheumatoid nodules to JAKi seen in our cohort is similar to that reported in some RA patients with pulmonary nodules.^5^^,^^10^^,^^11^

Although this represents the largest published experience of RA patients with cutaneous rheumatoid nodules who have responded to JAKi, this series remains limited by its small size and its noncontrolled, nonrandomized design. No men were included in this study, so we cannot comment on the effectiveness of this treatment in men.

Interferon-γ and IL-6, JAK1 signaling cytokines, have been implicated in the pathogenesis of RA and contribute to the formation of rheumatoid nodules,^12^ potentially through macrophage and T cell activation. These cytokines have also been found to be dysregulated in other granulomatous diseases characterized by aberrant mobilization of macrophages. As seen with rheumatoid nodules, JAKi have had efficacy in patients with the cutaneous granulomatous diseases, sarcoidosis, granuloma annulare, necrobiotic xanthogranuloma, and necrobiosis lipoidica.^13^^,^^14^

JAKi are uniquely effective in the treatment of active RA patients with rheumatoid nodules, likely through inhibition of pathogenic cytokine signaling that contributes to the development or maintenance of rheumatoid nodules that had not been targeted by previous therapies. This report offers strong support for the efficacy of JAKi for cutaneous rheumatoid nodules and provides additional rationale for their use in patients with other cutaneous granulomatous diseases.

## Conflicts of interest

Dr Kramer has stock shares of Abbvie and Pfizer. Drs Bulbin, Elliot Rosenstein, and Rachel Rosenstein have no conflicts of interest to declare.
